# *Teredinibacter haidensis* sp. nov., *Teredinibacter purpureus* sp. nov. and *Teredinibacter franksiae* sp. nov., marine, cellulolytic endosymbiotic bacteria isolated from the gills of the wood-boring mollusc *Bankia setacea* (Bivalvia: Teredinidae) and emended description of the genus *Teredinibacter*

**DOI:** 10.1099/ijsem.0.004627

**Published:** 2021-01-13

**Authors:** Marvin A. Altamia, J. Reuben Shipway, David Stein, Meghan A. Betcher, Jennifer M. Fung, Guillaume Jospin, Jonathan Eisen, Margo G. Haygood, Daniel L. Distel

**Affiliations:** ^1^​ Ocean Genome Legacy Center, Northeastern University, Nahant, MA, USA; ^2^​ The Centre for Enzyme Innovation, University of Portsmouth, Portsmouth, UK; ^3^​ Department of Microbiology, University of Massachusetts, Amherst, MA, USA; ^4^​ Downstream Strategies, Alderson, WV, USA; ^5^​ Perfect Day, Emeryville, CA, USA; ^6^​ College of Biological Sciences, University of California, Davis, CA, USA; ^7^​ Department of Medicinal Chemistry, College of Pharmacy, University of Utah, UT, USA

**Keywords:** conjugative elements, horizontal gene transfer, lateral gene transfer, lignocellulose degradation, nitrogen fixation, nitrate respiration, plasmid

## Abstract

Here, we describe three endosymbiotic bacterial strains isolated from the gills of the shipworm, *Bankia setacea* (Teredinidae: Bivalvia). These strains, designated as Bs08^T^, Bs12^T^ and Bsc2^T^, are Gram-stain-negative, microaerobic, gammaproteobacteria that grow on cellulose and a variety of substrates derived from lignocellulose. Phenotypic characterization, phylogeny based on 16S rRNA gene and whole genome sequence data, amino acid identity and percentage of conserved proteins analyses, show that these strains are novel and may be assigned to the genus *Teredinibacter *. The three strains may be differentiated and distinguished from other previously described *Teredinibacter* species based on a combination of four characteristics: colony colour (Bs12^T^, purple; others beige to brown), marine salt requirement (Bs12^T^, Bsc2^T^ and *Teredinibacter turnerae* strains), the capacity for nitrogen fixation (Bs08^T^ and *T. turnerae* strains) and the ability to respire nitrate (Bs08^T^). Based on these findings, we propose the names *Teredinibacter haidensis* sp. nov. (type strain Bs08^T^=ATCC TSD-121^T^=KCTC 62964^T^), *Teredinibacter purpureus* sp. nov. (type strain Bs12^T^=ATCC TSD-122^T^=KCTC 62965^T^) and *Teredinibacter franksiae* sp. nov. (type strain Bsc2^T^=ATCC TSD-123^T^=KCTC 62966^T^).

Cellulose is considered to be the most abundant biological material on Earth [1] and numerous organisms have exploited it for their nutritional needs [2]. It has been estimated that approximately 40 % of bacteria for which genome sequence data is available encode a copy of a putative cellulase gene; however, only around 4 % of these microbes are truly cellulolytic [3]. A number of cellulolytic bacteria form symbiotic partnerships with herbivorous animals. In terrestrial environments these animals include xylophagous insects such as termites and wood-eating roaches as well as a variety of herbivorous vertebrates, all of which harbour cellulolytic bacteria in their gut [4].

In marine environments, bivalves of the family *Teredinidae* (shipworms) are among the best-known hosts of cellulolytic bacteria [5]. Shipworms are worm-like wood-boring and wood-feeding bivalves that may be found in all oceans of the world, as well as in a variety of brackish to fresh water bays, rivers and estuaries [5]. Unlike the previously mentioned terrestrial animals, shipworms lack a conspicuous microbial community in the portion of the digestive tract in which wood digestion is thought to occur [6]. Instead, shipworms maintain dense populations of intracellular cellulolytic bacteria in specialized cells in their gills called bacteriocytes [7, 8]. Shipworm gill endosymbionts have been shown to secrete a variety of lignocellulolytic enzymes that are selectively transported to the cecum where they liberate simple sugars from the ingested wood that can readily be absorbed by the host. This form of nutritional symbiosis, wherein the principal enzyme producers are not in direct contact with the substrate, has only been observed in shipworms [9].

Shipworm gill endosymbionts are unique in that they are among the few intracellular endosymbionts that have been grown successfully in pure culture [10, 11]. To date, three of these endosymbiotic species have been formally described. Two of these, *Teredinibacter turnerae* and *Teredinibacter waterburyi* are microaerophilic cellulolytic bacteria placed within the family *Cellvibrionaceae* [11, 12]. The former is diazotrophic and is widely distributed within the members of the family *Teredinidae* [13], while the latter does not fix dinitrogen and has so far been found only in association with the feathery shipworm, *Bankia setacea* [12]. Consistent with their proposed function in supporting host xylotrophy, the genomes of these two bacterial species contain numerous genes encoding secreted carbohydrate-active enzymes (CAZymes) with predicted activities against plant cell-wall components [9, 14]. In addition to these cellulolytic endosymbionts, a sulfur-oxidizing chemoautotrophic, nitrogen-fixing endosymbiont, *Thiosocius teredinicola* 2141T^T^ has been isolated from the gills of the giant mud-dwelling shipworm *Kuphus polythalamius*, indicating that non-cellulolytic bacteria can also form symbiotic partnerships with teredinids [15, 16]

In addition to *T. waterburyi *, three similar endosymbiont strains, designated Bs08^T^, Bs12^T^ and Bsc2^T^ were isolated from feathery shipworm *Bankia setacea* [9]. Together these four strains were shown to encode a significant portion of the bacterial genes detected in the gill metagenome of this host species, signifying that these bacteria are the dominant endosymbionts in the *B. setacea* gill community [9]. In this study, we characterize these bacteria and propose the names *Teredinibacter haidensis* sp. nov., *Teredinibacter purpureus* sp. nov. and *Teredinibacter franksiae* sp. nov., with Bs08^T^, Bs12^T^ and Bsc2^T^, as their respective type strains.

## Isolation

Strains Bs08^T^, Bs12^T^ and Bsc2^T^ were isolated from the gill and cecum of the shipworm *B. setacea* collected from the Puget Sound, Washington State, USA as described by O’Connor *et al*. [9] and Altamia *et al*. [12]. Briefly, homogenates of gill or cecum tissues were streaked onto culture plates (motherplates) containing 1.0 % Bacto agar shipworm basal medium (SBM) [10] at pH 7.0 or 8.0 supplemented with 0.2 % w/v powdered cellulose (Sigmacell Type 101; Sigma-Aldrich) and 0.025 % NH_4_Cl. Plates were incubated at either 18 or 30 °C until individual colonies could be observed. To rapidly identify novel bacteria on the motherplates, 16S rRNA genes were amplified directly from the colonies using Phire Animal Tissue Direct PCR Kit (Finnzymes F-170S) using 27F (5′-AGAGTTTGATCMTGGCTCAG-3′) and 1492R (5′-TACGGYTACCTTGTTACGACTT-3′) primers [9] employing the manufacturer’s recommended protocol. Remnants of the colonies used for colony PCR were transferred to new plates and were further purified using at least two rounds of re-streaking to ensure clonal selection. On plates inoculated with either gill or cecum tissue homogenates colonies were initially slightly raised and translucent. However, upon prolonged incubation some colonies developed light brown or purple coloration.

Strain Bs08^T^ was isolated from a motherplate inoculated with gill homogenate and incubated at 30 °C. A purple colony growing on a motherplate incubated at 18 °C was purified and designated as strain Bs12^T^. Strain Bsc2^T^ was isolated from a motherplate inoculated with cecum homogenate and incubated at 18 °C.

The 16S rRNA gene of Bsc2^T^ is identical in sequence to that of symbiotic bacteria localized to the gills of *B. setacea* by metagenomic sequencing and fluorescent *in situ* hybridization [9] and to those previously amplified from gills by PCR [17]. Because Bsc2^T^ was shown to be abundant in gills of *B. setacea* but has not been detected in cecum by *in situ* hybridization techniques [9] and because the cecum of *B. setacea* has previously been shown to be largely devoid of bacteria [6], we suggest that the presence of strain Bsc2^T^ in cecum homogenates was likely due to contamination by neighbouring gill tissues during dissection.

For all the subsequent experiments, unless specified otherwise, strains were propagated in 6 ml of liquid SBM medium with either 0.2 % cellulose (w/v) or 0.5 % galactose (w/v) with 0.025 % NH_4_Cl in test tubes (18 mm × 150 mm) and shaken at 100 r.p.m. in an incubator set to 20 °C. Preparation and revival of frozen stocks are detailed in Altamia *et al*. [12].

The ability of the strains to utilize carbon substrates was tested by transferring colonies to SBM liquid medium supplemented with 0.025 % NH_4_Cl or 5 mM NaNO_3_ with the following carbon sources and concentrations (% w/v): polysaccharides—cellulose (0.2 %), carboxymethylcellulose (0.5 %), d-galacto-d-mannan from locust bean (0. 1%), β-d-glucan from barley (0.1 %), lichenan (0.1 %), starch (0.1 %), pectin (0.1 %), dextran (0.1 %), curdlan (0.1 %), pachyman (0.1 %), laminarin (0.1 %), pullulan (0.1 %), chitin (0.1%) and chitosan (0.1 %); sugars—cellobiose (0.5 %), xylan (0.5 %), sucrose (0.5 %), xylose (0.5 %) arabinose (0.5 %), galactose (0.5 %), glucose (0.5 %); organic acids—formate (0.1 %), acetate (0.1 %), propionate (0.1 %) and pyruvate (0.1 %); and amines—*N*-acetylglucosamine (0.1 %), gelatin (0.1 %), yeast extract (0.1 %) and casein hydrolysate (0.1 %). All strains grew on cellulose, galactomannan, β-d-glucan, lichenan, pullulan, gelatin and casein hydrolysate and all sugars tested. Strains Bs08^T^, Bs12^T^ and Bsc2^T^ and previously described *Teredinibacter* species do not hydrolyse agar, distinguishing them from the closely related *Saccharophagus degradans* 2-40^T^ [18] and *Agarilytica rhodophyticola* strain 017^T^ [19]. None of the tested strains grew on chitosan (see Table 1).

**Table 1.** Characteristics of strains Bs08^T^ *,* Bs12^T^, Bsc2^T^ and related type strains Strains: 1, Bs08^T^ (data from this study); 2, Bs12^T^ (data from this study); 3, Bsc2^T^ (data from this study) 4, *Teredinibacter waterburyi* Bs02^T^ [12]; 5, *Teredinibacter turnerae* T7902^T^ (data from this study and [11]); 6, *Saccharophagus degradans* 2-40^T^ [18]; 7, *Thalassocella blandensis* ISS155^T^ [33]; 8, *Agarilytica rhodophyticola* 017^T^ [19]. nr, Not reported; +, positive; −, negative.

Characteristic	1	2	3	4	5	6	7	8
Cell shape	Rods	Rods	Rods	Rods	Rods	Pleomorphic	Rods	Rods
Cell length (µm)	2–5	2–5	2–5	2–5	3–6	1–20	1.0–1.3	1.7–5.7
Habitat	Endosymbiont of *Bankia setacea*	Endosymbiont of *Bankia setacea*	Endosymbiont of *Bankia setacea*	Endosymbiont of *Bankia setacea*	Widely occurring shipworm endosymbiont	Free-living, isolated from *Spartina alterniflora*	Free-living, isolated from surface seawater	Free-living, isolated from *Glacilaria blodgettii*
Marine salt (Mg, Ca) requirement	−	+	+	−	+	+	+	nr
Microaerobic	+	+	+	+	+	nr	nr	nr
Optimum temperature (range) for growth (°C)	20 (15–30)	20 (10–25)	20 (15–20)	20 (15–30)	30–35 (~20–38)*	30 (4–37)	28–37 (15–37)	28–33 (15–40)
Optimum pH (range) for growth	8.0 (6.5–8.5)	8.0 (6.5–8.5)	8.0 (6.5–8.5)	8.0 (6.5–8.5)	8.5 (6.0–10.5)	7.5 (4.5–10.0)	7.0–8.0 (6.0–9.0)	8.0 (6.5–8.5)
Optimum salinity (range) for growth (M NaCl)	0.3 M (0.1–0.6 M)	0.4 M (0.2–0.6 M)	0.2 M (0.2–0.4 M)	0.5 M (0.0–0.8 M)	0.3 M (0.1–0.6 M)	0.6 M (0.2.–1.7 M)	0.4 M (0.3.–1.5 M)	0.5 M (0.3– 0.7 M)
Doubling time (h)	5.2	8.8	11.5	7.2	9.7^*^	nr	nr	nr
Vitamin requirement	−	−	−	−	−	B cofactors stimulatory	−	nr
Colony colour	Beige	Purple	Beige	Beige	Beige	nr	nr	nr
Nitrogen fixation	+	−	−	−	+	−	−	−
Nitrate respiration	+	−	−	−	−	nr	nr	−
Cellulose hydrolysis	+	+	+	+	+	+	+	−
Agar hydrolysis	−	−	−	−	−	+	−	+
0.1 % Starch	+	−	−	−	+	nr	nr	nr
0.1 % Pectin	+	+	+	+	+	nr	nr	nr
0.1 % Dextran	+	−	−	+	−	nr	nr	nr
0.1 % Curdlan	+	−	−	+	+	nr	nr	nr
0.1 % Pachyman	+	+	−	+	+	nr	nr	nr
0.1 % Laminarin	+	−	−	+	+	nr	nr	nr
0.1 % Chitin	+	−	−	+	+	nr	nr	nr
0.1 % *N*-Acetyl-d-glucosamine	+	−	−	+	+	nr	nr	nr
0.5 % Cellobiose	+	+	+	+	+	nr	−	nr
0.5 % Xylan	+	+	+	+	+	+	nr	nr
0.5 % Xylose	+	+	+	+	+	nr	−	nr
0.5 % Arabinose	+	+	+	+	nr	nr	nr	nr
0.5 % Galactose	+	+	+	+	nr	nr	−	+
0.5 % Glucose	+	+	+	+	+	+	+	nr
0.5 % Sucrose	+	+	+	+	+	nr	−	+
0.1 % Formate	−	−	−	−	nr	nr	nr	nr
0.1 % Acetate	−	−	−	−	+	nr	+	nr
0.1 % Propionate	−	−	−	−	nr	nr	−	nr
0.1 % Pyruvate	+	+	−	+	+	nr	nr	nr

*Data from [34].

The diazotrophic ability of these strains was tested by re-streaking colonies on SBM medium with 0.2 % cellulose but without an added nitrogen source. Of these three strains, only Bs08^T^ was found to be capable of nitrogen fixation. These results are supported by analyses of their respective genomes which indicate that only Bs08^T^ encodes genes of the Nif operon which is diagnostic of nitrogen fixation [9]. It has been shown that endosymbionts of the shipworm *Lyrodus pedicellatus* fix nitrogen *in situ* within host cells, suggesting symbiont diazotrophy might help supplement their nitrogen deficient diet of wood [20]. Interestingly, of the six shipworm gill endosymbionts described to date, three are diazotrophic (* T. turnerae* T7902^T^, Bs08^T^ and the thioautotrophic shipworm symbiont *Thiosocius teredinicola* 2141T^T^) and three (Bs02^T^, Bs12^T^ and Bsc2^T^) are incapable of fixing atmospheric dinitrogen. This indicates that although diazotrophy may be a common feature of shipworm gill endosymbionts, it is not a prerequisite for endosymbiotic existence. Notably, diazotrophy is not common among described species of the family *Cellvibrionaceae *. Of these, only *Cellvibrio diazotrophicus* [21] and *T. turnerae* [11] have been shown to fix nitrogen. Additionally, *nifH* homologs have been found in the genomes of *Agaribacterium haliotis* feces2^T^ and in the as-yet undescribed *Pseudomaricurvus* sp. HS19.

The ability of these three strains to respire nitrate was also tested. Briefly, cells grown in liquid SBM medium with 0.2 % cellulose and 5 mM NaNO_3_ were re-streaked on a solid version of this medium prepared with 1.0 % Bacto agar. Control plates containing SBM medium with 0.2 % cellulose and 0.0025 % NH_4_Cl were also used. The inoculated plates were then placed inside an anaerobic pouch (BS GasPak EZ Anaerobic Pouch). Despite the presence of homologs of dissimilatory nitrate reductase (*nar*) in the genomes of all three strains, only strain Bs08^T^ grew under anaerobic conditions in the presence of nitrate.

To determine the oxygen growth requirements of the three strains, stab cultures were prepared by inoculating soft agar tubes (18 mm×150 mm tubes containing 15 ml of 0.2 % Bacto agar (w/v), 1 x SBM medium, 0.2 % cellulose and 0.025 % NH_4_Cl) with liquid cultures using sterile needles. For all strains, a faint lens of cells forms a few millimetres below the soft agar surface, indicating that, like *T. turnerae* and *T. waterburyi* Bs02^T^, strains Bs08^T^, Bs12^T^ and Bsc2^T^, show increased growth under microaerobic conditions. Also, like *T, turnerae*, clearing of the medium was observed around the growth lens after prolonged incubation, due to the hydrolysis of cellulose.

Growth optima, ranges and doubling times were determined in an artificial seawater-based SBM liquid medium supplemented with 0.5 % galactose (w/v) and 0.025 % NH_4_Cl as previously described [12]. The data obtained for the three strains are shown in Table 1. Like *T. turnerae* T7902^T^, strains Bs12^T^ and Bsc2^T^, require concentrations of Ca^+2^ and Mg^+2^ in the medium similar to those found in seawater (~10 mM Ca and 50 mM Mg), while *T. waterburyi* Bs02^T^ and strain Bs08^T^ do not.

**Fig. 1.**
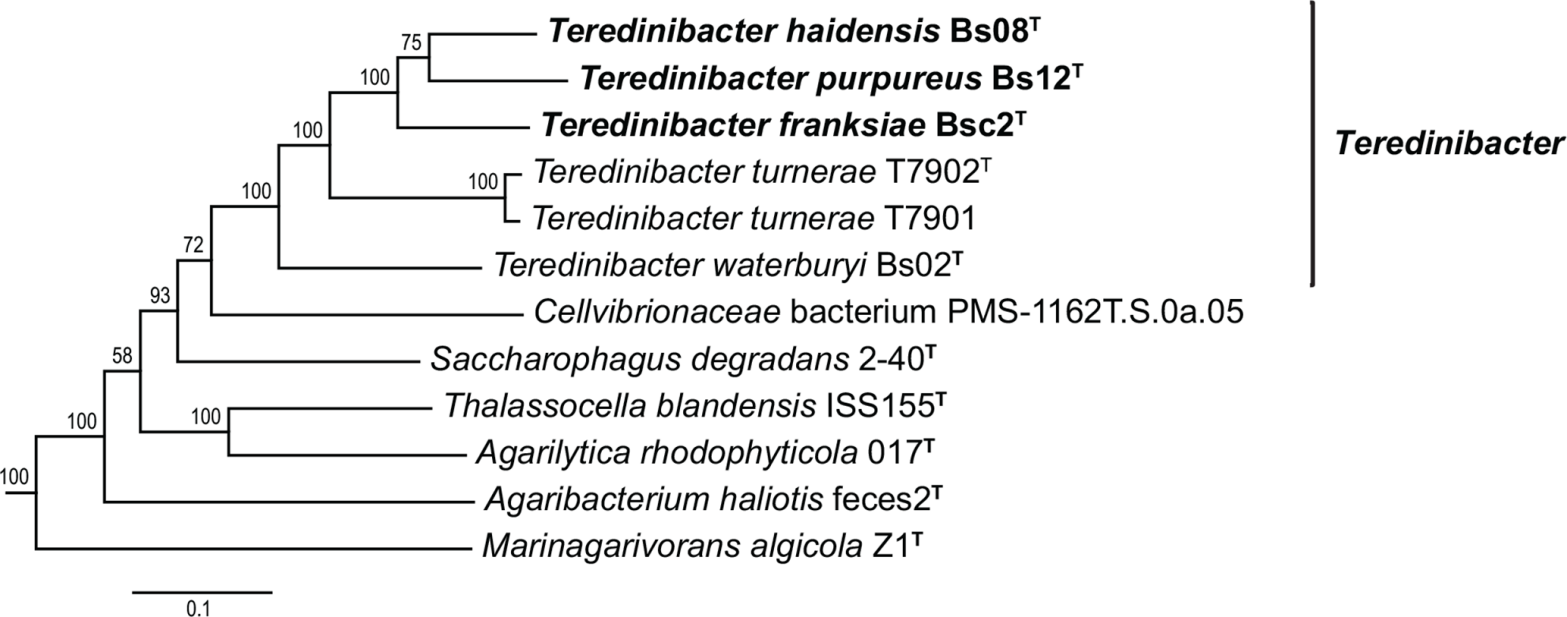
Phylogram depicting relationships among strains Bs08^T^, Bs12^T^ and Bsc2^T^ and related bacteria inferred by maximum-likelihood analysis based on multiple conserved protein-coding sequences. The tree was inferred using RaxML 8.2.12 with a concatenated amino acid sequence set including 120 conserved single-copy protein-coding marker genes identified in 58 genomes using the Genome Taxonomy Database Toolkit (GTDB-Tk 1.1.0). Values displayed at the nodes are percent bootstrap proportions of 250 replicates. The scale bar represents substitution rate per site. The figure presented here is a subtree excerpted from the larger tree with 61 taxa shown in Fig. S1.

Cellular fatty acid methyl ester (FAME) analysis was performed using the MIDI Sherlock Microbial Identification System. Briefly, week-old colonies were scraped from SBM medium agar plates supplemented with 0.5 % galactose and 0.025 % NH_4_Cl growing at 20 °C and submitted to midi (Newark, DE) for analyses. The three major fatty acids for all three strains are C_15 : 0_ iso, C_15 : 0_ anteiso and C_17 : 0_ anteiso. Strains Bs12^T^ and Bsc2^T^ have very similar FAME profiles wherein C_15 : 0_ anteiso and C_17 : 0_ anteiso account for more than 80 % of fatty acids detected. In strain Bs08^T^, the total for these two fatty acids is less than 50 %. FAME profiles of these three *B. setacea* endosymbionts are markedly different from recently described *B. setacea* symbiont *T. waterburyi* Bs02^T^ which has dominant fatty acids composed of summed feature 5 (C_18 : 0_ ante and/or C_18 : 2_ *ω6*,9*c*). This is consistent with its more divergent phylogenetic relationship as indicated in Fig. 1. A detailed comparison of the FAME profiles among all the shipworm symbionts and related bacteria is shown in Table S1 (available in the online version of this article).

## Morphology

## Full-Text

Colonies of strains Bs08^T^, Bs12^T^ and Bsc2^T^, growing on SBM plates supplemented with cellulose initially appear as minute, slightly raised and translucent. Upon prolonged incubation, the colonies widen in the subsurface of the agar and produce a characteristic clearing zone due to the hydrolysis of cellulose. At this stage, colonies of strains Bs08^T^ and Bsc2^T^ appear off-white or beige, while those of strain Bs12^T^ develop a deep-purple colour. Purple pigmentation is also observed in the culture supernatant when strain Bs12^T^ was grown in liquid medium. No similar pigment production was observed in Bs08^T^ and Bsc2^T^ cultures or in previously described *Teredinibacter* species. Morphology of cells at mid-exponential phase in liquid medium was examined using phase contrast light microscopy (Nikon Eclipse Ni-U and Nikon NIS Elements). The cells of all the three strains appear as motile slightly curved rods that are 2–5 µm long and 0.4–0.6 µm wide, and are Gram-stain negative (Sigma-Aldrich).

## Genomes

Genomic DNA extracts used for whole genome sequencing and 16S rRNA gene sequence amplification were obtained from bacterial cell pellets grown on SBM liquid medium with 0.2 % cellulose or 0.5 % galactose using the method described in O’Connor *et al*. [9] and Altamia *et al.* [12]. The genomes of strains Bs08^T^ and Bsc2^T^ were sequenced at New England Biolabs using PacBio Model RSII platform, while the genome of strain Bs12^T^ was sequenced by the Joint Genome Institute – Department of Energy (JGI-DOE) using PacBio Model RS II. All the genomes were assembled using HGAP version 2.0. Contigs from previously reported genome sequence data for these strains [9, 12] were aligned with the new assemblies and those determined to overlap multiple contigs were used to identify sequence gaps and the contig termini flanking each gap. Contiguity across identified gaps was then confirmed by PCR amplification and the sequence spanning each gap was determined from the resulting PCR products by primer walking using the Sanger sequencing method.

The assembled genome of strain Bs08^T^ is composed of a single circular chromosome of 4 915 572 bp with a G+C content of 47.2 mol%. The estimated sequencing coverage is 114×. The genome of strain Bsc2^T^ was assembled to form nine scaffolds totaling to 5 406 307 bp with a G+C content of 47.31 mol% with estimated coverage of 62×. The assembled genome of Bs12^T^ is composed of 2 circular elements, a chromosome of 4 643 613 bp with a DNA G+C content of 45.89 mol% and a large plasmid of 249 467 bp with a DNA G+C content of 42.54 mol%. Estimated genome coverage is 210×. The cumulative GC-skew plots calculated for the two elements are typical of closed-circular DNA structures [22] (data not shown). The smaller of the two, which was identified as a putative conjugative element using the ICEfinder [23] web-based detection tool (https://db-mml.sjtu.edu.cn/ICEfinder/ICEfinder.html), differs from the chromosome both in mol% G+C and in tetranucleotide frequency. Additionally, it contains a copy of *recA*, a gene most commonly found as a single copy in bacterial chromosomes [24]. A BLASTp search of the GenBank nr database (7/1/2020) indicates that this *recA* copy is most similar to that found in the chromosome of *Marinobacter confluentis *, sharing 79.52 % identity at 95 % query coverage with this *recA* as compared to 75.9 % identity at 95 % query coverage with the *recA* copy found in the Bs12^T^ chromosome. These data suggest that this plasmid was acquired recently via lateral gene transfer. Detailed analysis of the genomes of strains Bs08^T^, Bs12^T^ and Bsc2^T^ will be reported elsewhere. A table comparing the genomes of strains Bs08^T^, Bs12^T^ and Bsc2^T^ and related bacteria are shown in Table 2.

**Table 2.** Characteristics of the genomes of strains Bs08^T^, Bs12^T^, Bsc2^T^ and other related type strains Strain: 1, Bs08^T^ (data from this study); 2, Bs12^T^ (data from this study); 3, Bsc2^T^ (data from this study) 4, *Teredinibacter waterburyi* Bs02^T^ [12]; 5, *Teredinibacter turnerae* T7902^T^; 6, *Saccharophagus degradans* 2-40^T^ [18]; 7, *Thalassocella blandensis* ISS155^T^ [33]; 8, *Agarilytica rhodophyticola* 017^T^ [19]. +, Present; −, not present; nr, not reported.

Characteristic	1	2	3	4	5	6	7	8
Genome size (Mbp)	4.92	4.89	5.41*	4.03*	5.38	5.05	6.09	6.87*
G+C content (mol%)	47.2	45.7	47.3	47.7	50.8	45.8	40.95 %	40.2
Plasmids	−	1	−	−	−	−	−	3
Protein-coding genes	4119	4553	4624	3439	4425	4035	5425	5659
NifH cluster	+	−	−	−	+	−	−	−

*Genome not closed.

## Phylogeny

Phylogenetic analysis was carried out by aligning the near-full length 16S rRNA gene sequences of the three strains with related reference taxa using mafft version 7 with Q-INS-I [25] setting, followed by manual adjustments by eye. The final trimmed dataset is composed of 1372 nucleotide positions. Phylogenetic analysis was carried out using Bayesian inference (MrBayes version 3.2.6) [26]. Markov chain Monte Carlo chains were set to 5 million with subsampling every 2000 generations utilizing the GTR+I+Γ nucleotide substitution model, with the first 20  % of the results discarded as the analytical burn-in.

**Fig. 2.**
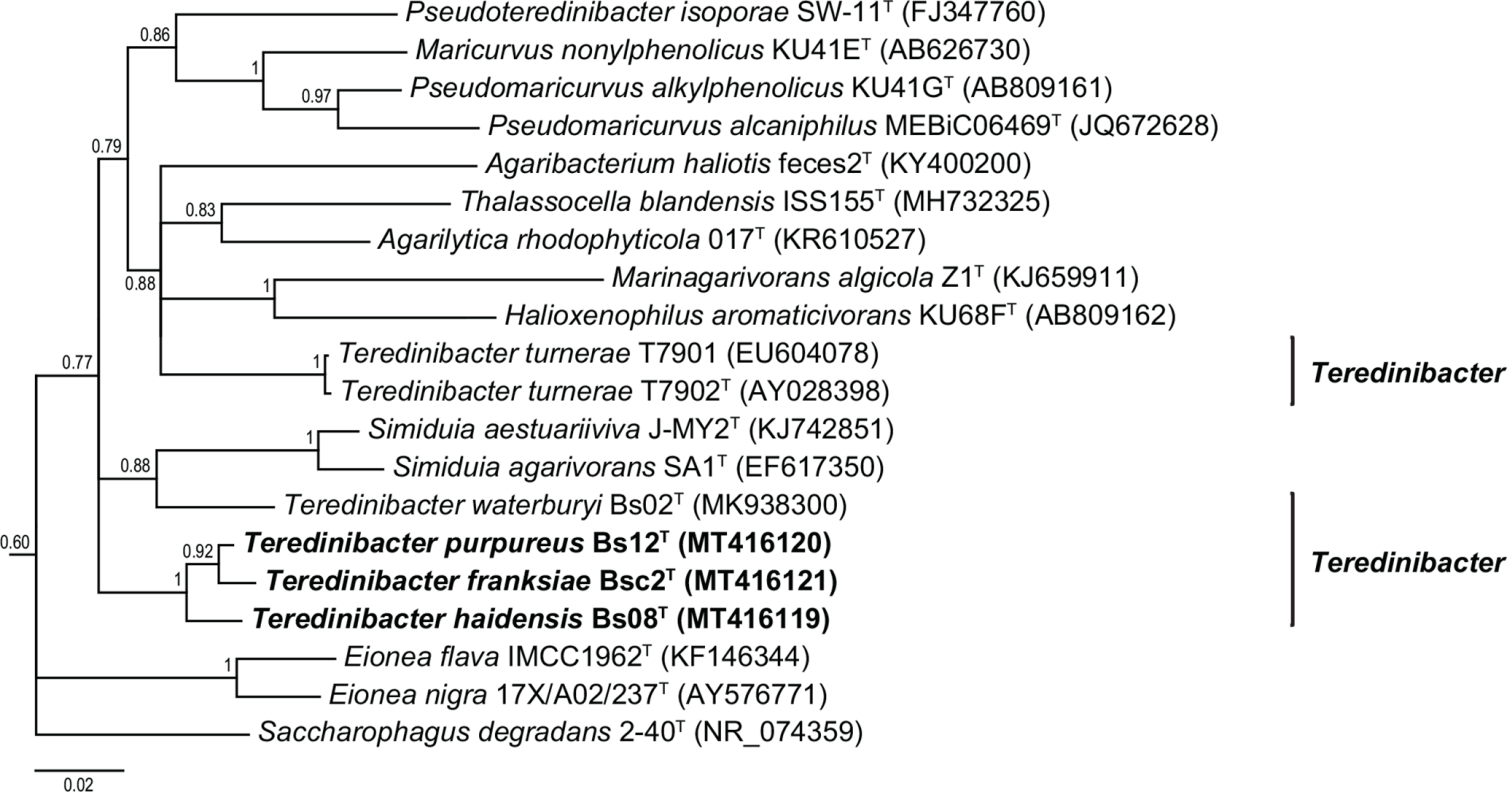
Phylogram depicting relationships among strains Bs08^T^, and Bsc2^T^ and related bacteria as inferred by Bayesian analysis of 16S rRNA gene sequences. The figure presented here is a subtree excerpted from the tree shown in Fig. S1, inferred using 1372 nucleotide positions employing GTR+I+Γ as the substitution model in MrBayes version 3.2.6. Chain length was set to 5 million, subsampling every 2000 generations and discarding the first 20 % of the analytical results as burn-in. Posterior probability values are indicated for each node. The scale bar represents nucleotide substitution rate per site.

Here and in previous analyses [12, 27], phylogenetic relationships among examined species within the family *Cellvibrionaceae* are poorly resolved based on analyses of 16S rRNA gene sequences alone (Figs 2 and S2). In these analyses, many nodes, including those associating the three new strains presented here with previously named taxa, display posterior probabilities below those commonly accepted as thresholds for significance. However, approaches that utilize concatenated alignments of multiple protein-coding loci have proven to produce more robust phylogenetic topologies and to more accurately delimit natural phylogenetic groups [27]. For this study, we utilized the Genome Taxonomy Database Toolkit (GTDB-Tk 1.1.0) [28] to construct a concatenated alignment of 120 conserved single copy protein coding marker genes from the genomes of Bs08^T^, Bs12^T^ and Bsc2^T^ and 58 selected reference taxa (Table S2) . Phylogenetic relationships among taxa were then inferred using RaxML 8.2.12 [29] employing the protgammablosum62 substitution matrix and 250 bootstrap replicates were performed to generate a consensus tree. The resulting topology provides strong support for a clade containing strains Bs08^T^, Bs12^T^ and Bsc2^T^ and previously described species of the genus *Teredinibacter *. The same analysis places *Cellvibrionaceae* bacterium PMS-1162T.S.0a.05 basal to this clade, although with more modest bootstrap support. (Figs 1 and S1). This as yet uncharacterized strain was isolated from a specimen of the shipworm *Lyrodus pedicellatus* collected in the Philippines.

Increasingly, genomic comparisons are being used to guide delimitation of species and genera. For example, average amino acid identity (AAI) values less than 85 and 60 % have been suggested as thresholds for delimitation of species and genera respectively [30]. Similarly, percent of conserved protein (POCP) values less than 50 % have been proposed as a threshold for differentiating species [31] Here, pairwise AAI and POCP values were calculated among shipworm symbionts and related taxa using the method described by Konstantinidis *et al.* [32] and Qin *et al.* [31] respectively. Pairwise AAI and POCP values observed among strains Bs08^T^, Bs12^T^, Bsc2^T^ and the described *Teredinibacter* species *T. waterburyi* Bs02^T^ and *T. turnerae* T7902^T^ range from 62.50–70.18 % (Table 3) and POCP values from 51.79–60.94 % (Table 4). These values support the proposed designation of these strains as distinct species within the genus *Teredinibacter *. In consideration of physiology, genome composition and phylogenetic relationships to described taxa, we propose the new names *Teredinibacter haidensis* sp. nov., *Teredinibacter purpureus* sp. nov. and *Teredinibacter franksiae* sp. nov., with Bs08^T^, Bs12^T^ and Bsc2^T^ as their respective type strains.

**Table 3.** Average amino acid identity matrix among strains Bs08^T^, Bs12^T^ and Bsc2^T^ and related bacteria Values shown are for two-way AAI calculated using the method of Konstantinidis *et al.* [32]. Colour scale is from red (highest) to green (lowest)

		1	2	3	4	5	6	7	8	9	10	11	12
**1**	*** Teredinibacter haidensis* Bs08^T^**		70.18	69.11	64.62	64.06	59.57	58.14	57.34	55.88	55.52	52.94	52.08
**2**	*** Teredinibacter purpureus* Bs12^T^**	70.12		67.67	64.16	62.83	59.09	57.78	56.62	55.41	55.35	52.68	51.69
**3**	*** Teredinibacter franksiae* Bsc2^T^**	69.11	67.67		62.95	63.12	58.42	57.95	56.42	55.38	54.97	52.22	51.48
**4**	*** Teredinibacter waterburyi* Bs02^T^**	64.62	64.16	62.95		62.50	59.95	58.81	57.60	56.53	55.93	53.72	53.17
**5**	*** Teredinibacter turnerae* T7902^T^**	64.06	62.83	63.12	62.50		57.91	57.85	56.65	54.89	55.23	52.53	51.55
**6**	*** Saccharophagus degradans* 2–40^T^**	59.57	59.09	58.42	59.95	57.91		58.32	56.82	55.79	55.40	53.19	51.82
**7**	*** Cellvibrionaceae* bacterium PMS-1162T.S.0a.05**	58.14	57.78	57.95	58.81	57.85	58.32		56.57	54.99	54.94	52.36	51.93
**8**	*** Thalassocella blandensis* ISS155^T^**	57.34	56.62	56.46	57.60	56.62	56.82	56.57		58.10	54.76	52.02	51.76
**9**	*** Agarilytica rhodophyticola* 017^T^**	55.88	55.41	55.38	56.53	54.89	55.79	54.99	58.10		54.43	51.30	51.03
**10**	*** Agaribacterium haliotis* feces2^T^**	55.52	55.35	54.97	55.93	55.23	55.40	54.99	54.76	54.43		52.10	51.52
**11**	*** Simiduia agarivorans* SA1^T^**	52.94	52.68	52.22	53.72	52.53	53.19	52.36	52.02	51.30	52.10		54.40
**12**	*** Pseudoteredinibacter isoporae* SW-11^T^**	52.08	51.69	51.48	53.17	51.55	51.82	51.93	51.76	51.03	51.52	54.40	

**Table 4.** Percentage of conserved protein matrix among strains Bs08^T^, Bs12^T^ and Bsc2^T^ and related bacteria Values shown were calculated using the method of Qin *et al.* [31]. Colour scale is from red (highest) to green (lowest).

		1	2	3	4	5	6	7	8	9	10	11	12
**1**	*** Teredinibacter haidensis* Bs08^T^**		58.18	60.94	59.36	59.10	52.59	50.47	41.48	38.03	46.88	41.49	34.38
**2**	*** Teredinibacter purpureus* Bs12^T^**	58.18		58.15	55.45	51.79	49.12	46.83	38.28	35.75	42.94	38.44	31.89
**3**	*** Teredinibacter franksiae* Bsc2^T^**	60.94	58.15		55.62	53.66	52.62	50.50	39.95	37.89	45.14	39.83	32.82
**4**	*** Teredinibacter waterburyi* Bs02^T^**	59.36	55.45	55.62		56.70	54.72	54.49	42.47	44.02	48.91	43.17	36.06
**5**	*** Teredinibacter turnerae* T7902^T^**	59.10	51.79	53.66	56.70		46.64	49.67	42.33	38.71	45.27	40.15	33.77
**6**	*** Saccharophagus degradans* 2–40^T^**	52.59	49.12	52.62	54.72	46.64		53.37	43.57	40.25	48.07	44.30	35.88
**7**	*** Cellvibrionaceae* bacterium PMS-1162T.S.0a.05**	50.47	46.83	50.50	54.49	49.67	53.37		42.01	38.47	45.98	41.32	35.20
**8**	*** Thalassocella blandensis* ISS155^T^**	41.48	38.28	39.95	42.47	42.33	43.57	42.01		40.85	39.44	38.47	31.99
**9**	*** Agarilytica rhodophyticola* 017^T^**	38.03	35.75	37.89	44.02	38.71	40.25	38.47	40.85		39.55	35.45	33.47
**10**	*** Agaribacterium haliotis* feces2^T^**	46.88	42.94	45.14	48.91	45.27	48.07	45.98	39.44	39.55		42.05	36.16
**11**	*** Simiduia agarivorans* SA1^T^**	41.49	38.44	39.83	43.17	40.15	44.30	41.32	38.47	35.45	42.05		43.96
**12**	*** Pseudoteredinibacter isoporae* SW-11^T^**	34.38	31.89	32.82	36.06	33.77	35.88	35.20	31.99	33.47	36.16	43.96	

## Emended description of the Genus *Teredinibacter* Distel et *al*. 2020

The description is as given by Distel et al. 2020 [12] with the following emendation. When grown on SBM agar plates supplemented with Sigmacell cellulose, mature colonies may be clear, white, yellow, beige, brown, or purple. The type species is *Teredinibacter turnerae *.

## Description of *Teredinibacter haidensis* sp. nov.

*Teredininbacter haidensis* (hai.den′sis. N.L. masc. adj. *haidensis* named in recognition of the students and faculty of the Hydaburg City School District and members of the Haida Nation, Hydaburg, Alaska for their contributions to the study of shipworm biology).

In addition to the characteristics of the genus, capable of fixing atmospheric dinitrogen under microaerophilic growth conditions. Can use NH_4_Cl or nitrate for growth under normoxic conditions. Has the ability to perform anaerobic nitrate respiration when grown in liquid or solid medium supplemented with cellulose as the sole carbon source. When grown on plates, small translucent colonies are initially observed on the agar surface, but over time the majority of growth forms an inverted dome of cells with off-white or beige coloration beneath the agar surface. The pH, temperature and salinity range for growth is approximately pH 6.5–8.5, 15–30 °C and 0.1–0.6 M NaCl, respectively; with optimum growth recorded at pH 8.0, 20 °C and 0.3 M NaCl. Under these conditions, the observed mean doubling time is approximately 5.2 h. The major fatty acids are C_15 : 0_ anteiso, C_15 : 0_ iso and C_17 : 0_ anteiso. The genome is estimated to be 4.92 Mbp. The G+C content is 47.2 mol%. The GenBank/EBML/DDBJ accession numbers for the bioprojects, chromosomal sequences and 16S rRNA genes for *Teredinibacter haidensis* sp. nov. Bs08^T^ are PRJNA340180, CP060084 and MT416119, respectively. The type strain has been deposited as *T. haidensis* Bs08^T^ (=ATCC TSD-121^T^=KCTC 62964^T^).

## Description of *Teredinibacter purpureus* sp. nov.

*Teredininbacter purpureus* (pur.pu′re.us. L. masc. adj. *purpureus* purple).

In addition to the characteristics of the genus, requires a source of combined nitrogen such as NH_4_Cl or nitrate for growth. When grown on plates, small translucent colonies are initially observed on the agar surface, but over time the majority of growth forms an inverted dome of cells with pronounced purple coloration beneath the agar surface. The pH, temperature and salinity range for growth are approximately pH 6.5–8.5, 10–25 °C and 0.2–0.6 M NaCl, respectively; with the optimum growth recorded at pH 8.0, 20 °C and 0.4 M NaCl. Under these conditions, the mean doubling time is approximately 8.8 h. The major fatty acids are C_15 : 0_ anteiso, C_17 : 0_ anteiso and C_15 : 0_ iso. The total genome is estimated to be 4.89 Mbp and is composed of two closed circular elements, a chromosome of 4 643 613 bp with a DNA G+C content of 45.89 mol% and a large plasmid of 249 483 bp with a DNA G+C content of 42.54 mol%. The GenBank/EBML/DDBJ accession numbers for the bioproject, chromosomal sequence, plasmid sequence and 16S rRNA genes for *Teredinibacter purpureus* sp. nov. Bs12^T^ are PRJNA195847, CP060092, CP060093 and MT416120, respectively. The type strain has been deposited as *T. purpureus* Bs12^T^ (=ATCC TSD-122^T^=KCTC 62965^T^).

## Description of *Teredinibacter franksiae* sp. nov.

*Teredininbacter franksiae* (frank′si.ae. N.L. gen. n. *franksiae* named in honour of microbiologist Diana Franks for her contributions to the characterization of the genus *Teredinibacter *).

In addition to the characteristics of the genus, requires a source of combined nitrogen such as NH_4_Cl or nitrate for growth. When grown on plates, small translucent colonies are initially observed on the agar surface, but over time the majority of growth forms an inverted dome of cells with off-white or beige coloration beneath the agar surface. The pH, temperature and salinity range for growth is approximately pH 6.5–8.5, 15–20 °C and 0.2–0.4 M NaCl, respectively; with the optimum growth recorded at pH 8.0, 20 °C and 0.2 M NaCl. Under these conditions, the mean doubling time is approximately 11.5 h. The major fatty acids are C_15 : 0_ anteiso, C_17 : 0_ anteiso and C_15 : 0_ iso. The total genome is estimated to be 5.41 Mbp with a G+C content of 47.3 mol%. The GenBank/EBML/DDBJ accession numbers for the bioprojects, chromosomal sequences, 16S rRNA genes for *Teredinibacter franksiae* sp. nov. Bsc2^T^ are PRJNA655743, JACJUV000000000, and MT416121, respectively. The type strain has been deposited as *T. franksiae* Bcs2^T^ (=ATCC TSD-123^T^=KCTC 62966^T^).

## Supplementary Data

Supplementary material 1

## References

[1] Duchesne LC, Larson DW (1989). Cellulose and the evolution of plant life. Bioscience.

[2] Cragg SM, Beckham GT, Bruce NC, Bugg TD, Distel DL (2015). Lignocellulose degradation mechanisms across the tree of life. Curr Opin Chem Biol.

[3] Koeck DE, Pechtl A, Zverlov VV, Schwarz WH, Pechtl A, Zverlov VV (2014). Genomics of cellulolytic bacteria. Curr Opin Biotechnol.

[4] Haigler CH, Weimer PJ (1991). Biosynthesis and Biodegradation of Cellulose.

[5] Distel DL, Goodell B, Nicholas DD, Schultz TP (2003). The biology of marine wood boring bivalves and their bacterial endosymbionts. Wood Deterioration and Preservation (ACS Symposium Series).

[6] Betcher MA, Fung JM, Han AW, O'Connor R, Seronay R (2012). Microbial distribution and abundance in the digestive system of five shipworm species (Bivalvia: Teredinidae). PLoS One.

[7] Popham JD, Dickson MR (1973). Bacterial associations in the teredo *Bankia australis* (Lamellibranchia: Mollusca). Mar Biol.

[8] Distel DL, DeLong EF, Waterbury JB (1991). Phylogenetic characterization and in situ localization of the bacterial symbiont of shipworms (*Teredinidae: Bivalvia*) by using 16S rRNA sequence analysis and *oligodeoxynucleotide* probe hybridization. Appl Environ Microbiol.

[9] O'Connor RM, Fung JM, Sharp KH, Benner JS, McClung C (2014). Gill bacteria enable a novel digestive strategy in a wood-feeding mollusk. Proc Natl Acad Sci U S A.

[10] Waterbury JB, Calloway CB, Turner RD (1983). A cellulolytic nitrogen-fixing bacterium cultured from the gland of deshayes in shipworms (Bivalvia: Teredinidae). Science.

[11] Distel DL, Morrill W, MacLaren-Toussaint N, Franks D, Waterbury J (2002). *Teredinibacter turnerae* gen. nov., sp. nov., a dinitrogen-fixing, cellulolytic, endosymbiotic gamma-proteobacterium isolated from the gills of wood-boring molluscs (Bivalvia: Teredinidae). Int J Syst Evol Microbiol.

[12] Altamia MA, Shipway JR, Stein D, Betcher MA, Fung JM (2020). *Teredinibacter waterburyi* sp. nov., a marine, cellulolytic endosymbiotic bacterium isolated from the gills of the wood-boring mollusc *Bankia setacea* (Bivalvia: Teredinidae) and emended description of the genus *Teredinibacter*. Int J Syst Evol Microbiol.

[13] Altamia MA, Wood N, Fung JM, Dedrick S, Linton EW (2014). Genetic differentiation among isolates of *Teredinibacter turnerae,* a widely occurring intracellular endosymbiont of shipworms. Mol Ecol.

[14] Yang JC, Madupu R, Durkin AS, Ekborg NA, Pedamallu CS (2009). The complete genome of *Teredinibacter turnerae* T7901: an intracellular endosymbiont of marine wood-boring bivalves (shipworms). PLoS One.

[15] Altamia MA, Shipway JR, Concepcion GP, Haygood MG, Distel DL (2019). *Thiosocius teredinicola* gen. nov., sp. nov., a sulfur-oxidizing chemolithoautotrophic endosymbiont cultivated from the gills of the giant shipworm, *Kuphus polythalamius*. Int J Syst Evol Microbiol.

[16] Distel DL, Altamia MA, Lin Z, Shipway JR, Han A (2017). Discovery of chemoautotrophic symbiosis in the giant shipworm *Kuphus polythalamia* (Bivalvia: Teredinidae) extends wooden-steps theory. Proc Natl Acad Sci U S A.

[17] Sipe AR, Wilbur AE, Cary SC (2000). Bacterial symbiont transmission in the wood-boring shipworm *Bankia setacea* (Bivalvia: Teredinidae). Appl Environ Microbiol.

[18] Ekborg NA, Gonzalez JM, Howard MB, Taylor LE, Hutcheson SW (2005). *Saccharophagus degradans* gen. nov., sp. nov., a versatile marine degrader of complex polysaccharides. Int J Syst Evol Microbiol.

[19] Ling SK, Xia J, Liu Y, Chen GJ, Du Z-J (2017). *Agarilytica rhodophyticola* gen. nov., sp. nov., isolated from *Gracilaria blodgettii*. Int J Syst Evol Microbiol.

[20] Lechene CP, Luyten Y, McMahon G, Distel DL (2007). Quantitative imaging of nitrogen fixation by individual bacteria within animal cells. Science.

[21] Suarez C, Ratering S, Kramer I, Schnell S (2014). *Cellvibrio diazotrophicus* sp. nov., a nitrogen-fixing bacteria isolated from the rhizosphere of salt meadow plants and emended description of the genus Cellvibrio. Int J Syst Evol Microbiol.

[22] Grigoriev A (1998). Analyzing genomes with cumulative skew diagrams. Nucleic Acids Res.

[23] Liu M, Li X, Xie Y, Bi D, Sun J (2019). ICEberg 2.0: an updated database of bacterial integrative and conjugative elements. Nucleic Acids Res.

[24] Lin Z, Kong H, Nei M, Ma H (Research Support, N.I.H., Extramural Research Support, Non-U.S. Gov't 2006). Origins and evolution of the recA/RAD51 gene family: evidence for ancient gene duplication and endosymbiotic gene transfer. Proc Natl Acad Sci U S A.

[25] Katoh K, Misawa K, Kuma K, Miyata T (2002). MAFFT: a novel method for rapid multiple sequence alignment based on fast Fourier transform. *Nucleic acids research*, Comparative Study Research Support, Non-U. S. Gov't.

[26] Ronquist F, Huelsenbeck JP (2003). MrBayes 3: Bayesian phylogenetic inference under mixed models. Bioinformatics.

[27] Spring S, Scheuner C, Goker M, Klenk HP (2015). A taxonomic framework for emerging groups of ecologically important marine Gammaproteobacteria based on the reconstruction of evolutionary relationships using genome-scale data. Front Microbiol.

[28] Chaumeil PA, Mussig AJ, Hugenholtz P, Parks DH (2019). GTDB-Tk: a toolkit to classify genomes with the genome taxonomy database. Bioinformatics.

[29] Stamatakis A (2014). RAxML version 8: a tool for phylogenetic analysis and post-analysis of large phylogenies. Bioinformatics.

[30] Rodriguez-R LM, Konstantinidis KT (2014). Bypassing cultivation to identify bacterial species. Microbe.

[31] Qin QL, Xie BB, Zhang XY, Chen XL, Zhou BC (2014). A proposed genus boundary for the prokaryotes based on genomic insights. J Bacteriol.

[32] Konstantinidis KT, Tiedje JM (2005). Towards a genome-based taxonomy for prokaryotes. J Bacteriol.

[33] Lucena T, Arahal DR, Sanz-Saez I, Acinas SG, Sánchez O (2020). *Thalassocella blandensis* gen. nov., sp. nov., a novel member of the family *Cellvibrionaceae*. Int J Syst Evol Microbiol.

[34] Greene RV, Freer SN (1986). Growth characteristics of a novel nitrogen-fixing cellulolytic bacterium. Appl Environ Microbiol.

