# Supplementary material for: Teredinibacter haidensis sp. nov., Teredinibacter purpureus sp. nov. and Teredinibacter franksiae sp. nov., marine, cellulolytic endosymbiotic bacteria isolated from the gills of the wood-boring mollusc Bankia setacea (Bivalvia: Teredinidae) and emended description of the genus Teredinibacter
Source: Int J Syst Evol Microbiol. 2021 Jan 13;71(2):004627. doi: 10.1099/ijsem.0.004627 (PMC8346767; doi:10.1099/ijsem.0.004627)
Supplement: Supplementary material 1 [file ijsem-71-627-s001.pdf]

Supplemental Figures and Tables

Teredinibacter haidensis sp. nov., *Teredinibacter purpureus* sp. nov., and *Teredinibacter franksiae* sp. nov., marine, cellulolytic endosymbiotic bacteria isolated from the gills of the wood-boring mollusc *Bankia setacea* (Bivalvia: Teredinidae) and emended description of the genus *Teredinibacter*

Marvin A. Altamia, J. Reuben Shipway, David P. Stein, Meghan A. Betcher, Jennifer M. Fung, Guillaume Jospin, Jonathan A. Eisen, Margo G. Haygood, Daniel L. Distel

International Journal of Systematic and Evolutionary Microbiology

Corresponding author: Daniel L. Distel
Ocean Genome Legacy Center
Northeastern University
430 Nahant Road
Nahant, MA, USA
email: d.distel@northeastern.edu

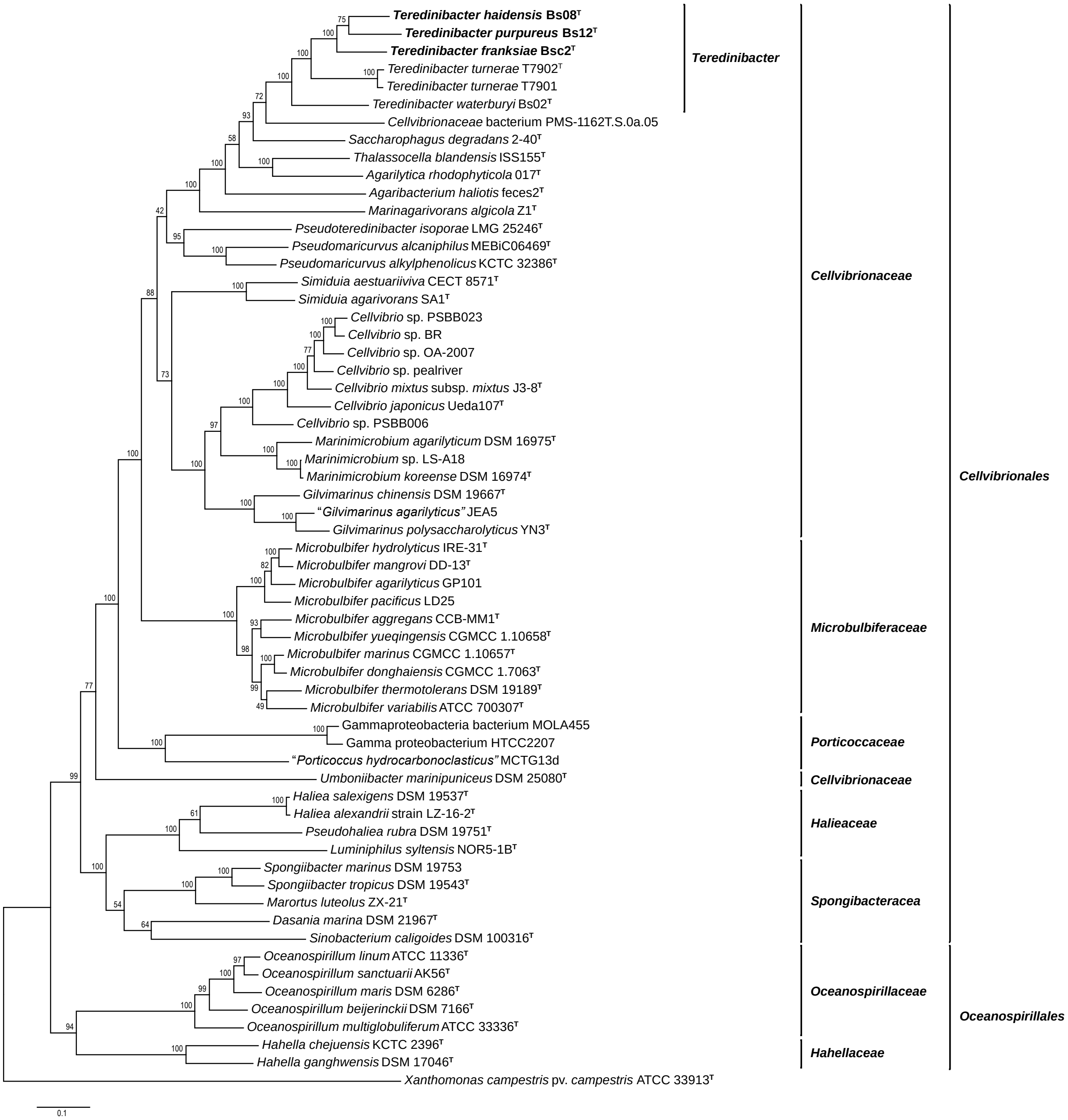

Figure S1: Phylogram depicting relationships among *T. haidensis* Bs08^T, *T. purpureus* Bs12^T, *T. franksiae* Bsc2^T, and related bacteria. The tree was inferred using RaxML 8.2.12 with a concatenated amino acid sequence set including 120 conserved single-copy protein-coding marker genes identified in 61 genomes using the Genome Taxonomy Database Toolkit (GTDB-Tk 1.1.0). Values displayed at the nodes are percent bootstrap proportions of 250 replicates. The scale bar represents substitution rate per site. A subtree excerpted from this tree is shown in Figure 1.

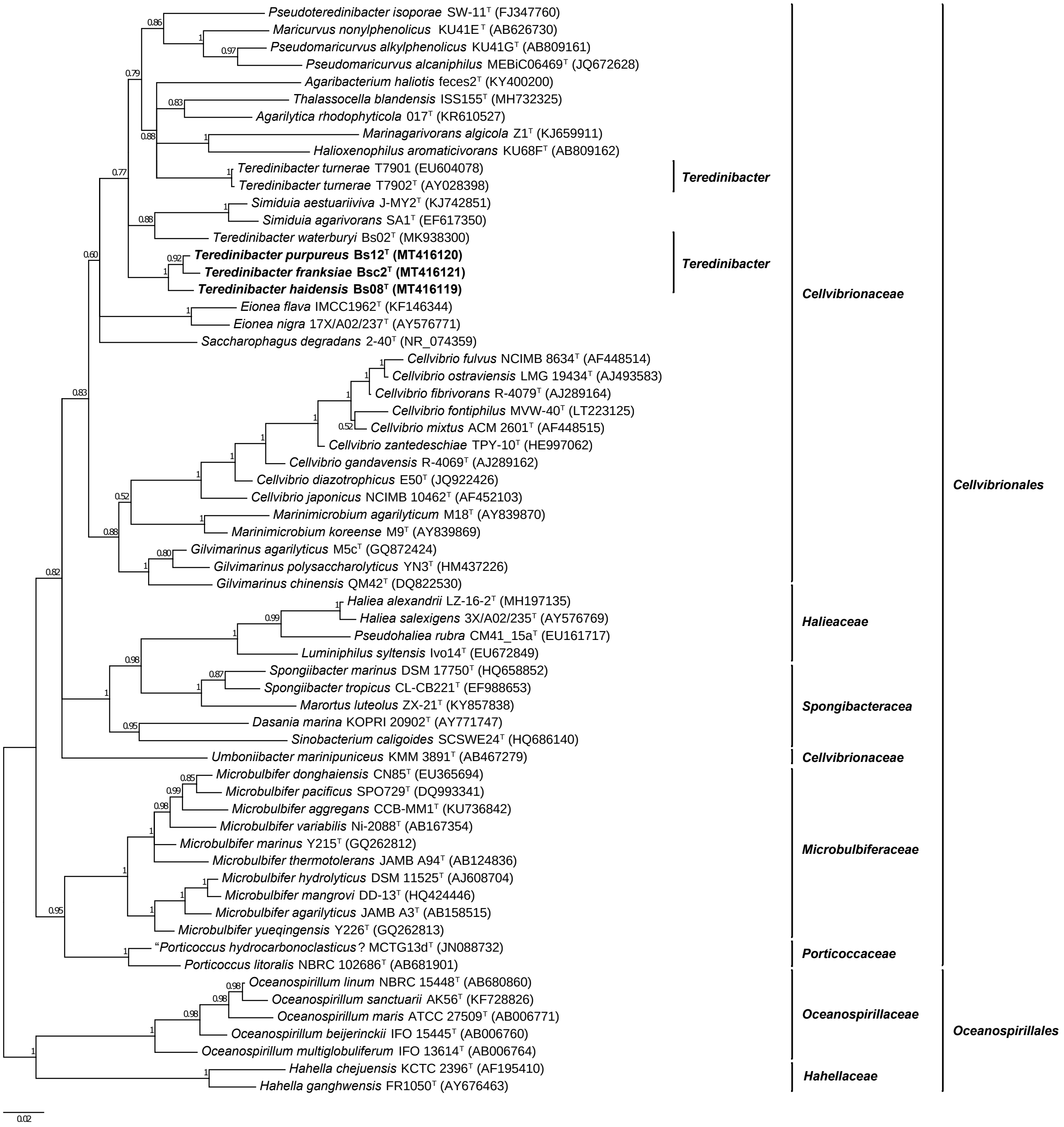

Figure S2: Phylogram depicting relationships inferred among *T. haidensis* Bs08^{T}, *T. purpureus* Bs12^{T}, and *T. franksiae* Bsc2^{T}, and related bacteria based on 16S rRNA sequences. The tree was constructed using an alignment of 1,372 nucleotide positions employing GTR+I+Γ as the substitution model in MrBayes version 3.2.6. Chain length was set to 5 million, subsampling every 2,000 generations and discarding the first 20% of the analytical results as burn-in. Posterior probability values are indicated for each node. The scale bar represents nucleotide substitution rate per site. A subtree excerpted from this tree is shown in Figure 2.

Table S1. Cellular fatty acid contents (percentage) of **1**, *T. haidensis* strain Bs08^{T}; **2**, *T. purpureus* strain Bs12^{T}; **3**, *T. franksiae* strain Bsc2^{T} and **4**, *T. waterburyi* strain Bs02^{T}. Tr denotes traces (<0.5%). The three most abundant fatty acids in each type strain are in boldface. Summed features are peaks consisting of two or more fatty acid methyl esters that that cannot be separated in MIDI system. Summed feature 2: $C_{14:0}$ 3-OH and/or $C_{16:1}$ iso; Summed feature 3: $C_{16:1}$ *ω*7*c* and/or $C_{15:0}$ iso 2-OH; Summed feature 4: $C_{17:1}$ isol I/anteiso B; Summed feature 5: $C_{18:0}$ ante and/or $C_{18:2}$ *ω*6,9*c*.

**Fatty acids**	**1**	**2**	**3**	**4**
$C_{9:0}$				0.9
$C_{10:0}$				
$C_{11:0}$				
$C_{10:0}$ 3-OH				
Unknown 11.799				
$C_{12:0}$				1.1
$C_{12:1}$ 3-OH				
$C_{12:0}$ 2-OH				
$C_{12:0}$ 3-OH				
$C_{13:0}$ anteiso		0.2	0.2	
$C_{14:0}$	0.3	0.2	0.2	1.0
$C_{14:0}$ iso	0.7	0.2	0.2	
$C_{15:0}$				
$C_{15:0}$ iso	**29.7**	**5.0**	**5.1**	
$C_{15:0}$ anteiso	**33.8**	**55.5**	**57.6**	
$C_{15:1}$ anteiso A		0.1		
$C_{16:0}$	1.6	0.9	0.8	**13.4**
$C_{16:0}$ iso	2.6	2.0	2.1	
$C_{16:0}$ iso 3-OH				
$C_{16:1}$ iso G		0.5	0.5	
$C_{16:1}$ ω7*c*-OH	1.1			
$C_{16:1}$ ω11*c*-OH	1.1			
$C_{17:0}$ iso				
$C_{17:0}$ iso	8.8	1.2	1.2	
$C_{17:0}$ anteiso	**14.4**	**31.3**	**29.4**	
$C_{17:0}$ 2-OH				0.6
$C_{17:1}$ ω8*c*				0.4
$C_{17:1}$ ω9*c*		2.8	2.9	
$C_{17:1}$ ω10*c*	2.1			
$C_{18:1}$ ω9*c*	0.6	0.2		**10.3**
$C_{18:1}$ ω7*c*				3.0
$C_{18:1}$ ω6*c*				
$C_{18:0}$				2.3
$C_{19:0}$ iso				0.4
Summed feature 3*	0.8			7.7
Summed feature 4*	2.5			
Summed feature 5*				**59.0**
Total	100.0	100.0	100.0	100.0

**Table S2: Whole genome sequence assemblies used to construct a protein-based phylogenetic tree.**		
	**Species name**	**Whole genome assembly accession**
1	*Teredinibacter haidensis* Bs08^T	GenBank GCF_014211975.1
2	*Teredinibacter purpureus* Bs12^T	GenBank GCA_014217335.1
3	*Teredinibacter franksiae* Bsc2^T	GenBank GCF_014218805.1
4	*Teredinibacter waterburyi* Bs02^T	GenBank GCF_001922985.1
5	*Teredinibacter turnerae* T7902^T	GenBank GCF_000379165.1
6	*Teredinibacter turnerae* T7901	GenBank GCF_000023025.1
7	*Agaribacterium haliotis* feces2^T	GenBank GCF_002312815.1
8	*Agarilytica rhodophyticola* 017^T	GenBank GCF_002157225.2
9	*Cellvibrio japonicus* Ueda107^T	GenBank GCF_000019225.1
10	*Cellvibrio mixtus* subsp. *mixtus* J3-8^T	GenBank GCF_000766945.1
11	*Cellvibrio* sp. BR	GenBank GCF_000263355.1
12	*Cellvibrio* sp. OA-2007	GenBank GCF_000953825.1
13	*Cellvibrio* sp. pearlriver	GenBank GCF_001183545.1
14	*Cellvibrio* sp. PSB006	GenBank GCF_002162135.1
15	*Cellvibrio* sp. PSBB023	GenBank GCF_002007605.1
16	*Cellvibrionaceae* bacterium PMS-1162T.S.0a.05	GenBank GCF_000964245.1
17	*Dasania marina* DSM 21967^T	GenBank GCF_000373485.1
18	Gammaproteobacterium HTCC2207	GenBank GCF_000153445.1
19	Gammaproteobacterium MOLA455	GenBank GCF_000511875.1
20	*"Gilvimarinus agarilyticus"* JEA5	GenBank GCF_000832015.1
21	*Gilvimarinus chinensis* DSM 19667^T	GenBank GCF_000377745.1
22	*Gilvimarinus polysaccharolyticus* YN3^T	GenBank GCF_001187555.1
23	*Hahella chejuensis* KCTC 2396^T	GenBank GCF_000012985.1
24	*Hahella ganghwensis* DSM 17046^T	GenBank GCF_000376785.1
25	*Haliea alexandrii* LZ-16-2^T	GenBank GCF_000423125.1
26	*Haliea salexigens* DSM 19537^T	GenBank GCF_900100355.1

27	*Luminiphilus syltensis* NOR5-1B^{T}	GenBank GCF_000158175.1
28	*Marinagarivorans algicola* Z1^{T}	GenBank GCF_001292705.1
29	*Marinimicrobium agarilyticum* DSM 16975^{T}	GenBank GCF_000423345.1
30	*Marinimicrobium koreense* DSM 16974^{T}	GenBank GCF_003762925.1
31	*Marinimicrobium* sp. LS-A18	GenBank GCF_000463525.1
32	*Marortus luteolus* ZX-21^{T}	GenBank GCF_002915595.1
33	*Microbulbifer agarilyticus* GP101	GenBank GCF_001999945.1
34	*Microbulbifer aggregans* CCM-MM1^{T}	GenBank GCF_001750105.1
35	*Microbulbifer donghaiensis* CGMCC 1.7063^{T}	GenBank GCF_900129095.1
36	*Microbulbifer hydrolyticus* IRE-31^{T}	GenBank GCF_009931115.1
37	*Microbulbifer mangrovi* DD-13^{T}	GenBank GCF_002009015.1
38	*Microbulbifer marinus* CGMCC 1.10657^{T}	GenBank GCF_900107725.1
39	*Microbulbifer pacificus* LD25	GenBank GCF_002959965.1
40	*Microbulbifer thermotolerans* DSM 19189^{T}	GenBank GCF_900112305.1
41	*Microbulbifer variabilis* ATCC 700307^{T}	GenBank GCF_000380565.1
42	*Microbulbifer yueqingensis* CGMCC 1.10658^{T}	GenBank GCF_900100355.1
43	*Oceanospirillum beijerinckii* DSM 7166^{T}	GenBank GCF_000422425.1
44	*Oceanospirillum linum* ATCC 11336^{T}	GenBank GCF_001995095.2
45	*Oceanospirillum maris* DSM 6286^{T}	GenBank GCF_000422865.1
46	*Oceanospirillum multiglobuliferum* ATCC 33336^{T}	GenBank GCF_900167095.1
47	*Oceanospirillum sanctuarii* AK56^{T}	GenBank GCF_002150805.1
48	*"Porticoccus hydrocarbonoclasticus"* MCTG13d	GenBank GCF_000744735.1
49	*Pseudohaliea rubra* DSM 19751^{T}	GenBank GCF_000764025.1
50	*Pseudomaricurvus alcaniphilus* MEBiC06469^{T}	GenBank GCF_011440395.1
51	*Pseudomaricurvus alkylphenolicus* KCTC 32386^{T}	GenBank GCF_011683955.1
52	*Pseudoteredinibacter isoporae* LMG 25246^{T}	GenBank GCF_011602085.1
53	*Saccharophagus degradans* 2-40^{T}	GenBank GCF_000013665.1
54	*Simiduia aestuariiviva* CECT 8571^{T}	IMG/MER 2824476646
55	*Simiduia agarivorans* SA1^{T}	GenBank GCF_000305785.2

56	*Sinobacterium caligoides* DSM 100316^{T}	GenBank GCF_003752585.1
57	*Spongiibacter marinus* DSM 19753	GenBank GCF_000422345.1
58	*Spongiibacter tropicus* DSM 19543^{T}	GenBank GCF_000420325.1
59	*Thalassocella blandensis* ISS155^{T}	GenBank GCF_902141825.1
60	*Umboniibacter marinipuniceus* DSM 25080^{T}	GenBank GCF_003688415.1
61	*Xanthomonas campestris* pv. *campestris* ATCC 33913^{T}	GenBank GCF_000007145.1

Supplemental scripts

```
gtdbtk classify_wf --genome_dir

--out_dir

GTDB-Tk --prefix Bankia_all2020 -x .fna --cpus 24


raxmlHPC-PTHREADS -m PROTGAMMABLOSUM62 -x 47 -p 8 -T 24 -N 250 -f a -s
Bankia_all2020.bac120.user_msa.fasta -n Bankia_all_GTDBtk
```
